# Health care professionals’ adherence to partograph use in Ethiopia: analysis of 2016 national emergency obstetric and newborn care survey

**DOI:** 10.1186/s12884-020-03344-6

**Published:** 2020-10-23

**Authors:** Solomon Weldemariam Gebrehiwot, Mulugeta Woldu Abrha, Haftom Gebrehiwot Weldearegay

**Affiliations:** 1 MekelleUniversity, College of Health Sciences, Mekelle, Ethiopia; 2 Tigray Health Research Institute, Mekelle, Ethiopia

**Keywords:** Adherence, Partograph, Health care providers, Emergency obstetrics care, Ethiopia

## Abstract

**Background:**

The period around childbirth and the first 24 hours postpartum remains a perilous time for both mother and newborn. Health care providers’ compliance to the World Health Organization modified partogram across the active first stage of labor is a graphic representation of a mother’s condition that is used as a guide in providing quality obstetrics care. However, little evidence is documented on the health providers’ adherence to the use of the partograph in Ethiopia, which limits health care providers’ ability to improve quality care services. Therefore, this study assessed the adherence of partograph use and associated factors in Ethiopia.

**Methods:**

Data from the Ethiopian 2016 National Emergency Obstetric and Newborn Care survey of 3,804 health facilities that provided maternity services were used. We extracted 2611 partograph charts over a 12 months period prior to the survey to review the proper recording of each component. Data analyses were performed using SPSS version 22.0 software. A logistic regression analyses was used to identify the association of explanatory variables with the outcome variable. A p-value of <0.05 was considered as cut off point to declare the significance association in the multivariable analysis.

**Results:**

Of the total 2611 partographs reviewed, 561(21.5%) of them were fully recorded as per the WHO guideline. Particularly, molding in 50%, color of liquor in 70.5%, fetal heart beat in 93.3%, cervical dilation in 89.6%, descent in 63.2%, uterine contraction in 94.5%, blood pressure in 80.5%, pulse rate in 70.5%, and temperature in 53% were accurately recorded. The odds of adherence to partograph use were 1.4 in rural health facilities when compared to their counterparts (AOR=1.44; 95% CI: 1.15, 1.80, *P*- 0.002).

**Conclusion:**

This study revealed a poor level of adherence in partograph use in Ethiopia. Molding, maternal temperature and decent were the least recorded parameters of the partograph. The odds of completion of partograph were high in rural facilities. Strong supporting supervision and mentoring the health workers to better record and use of partograph are needed mainly in urban health facilities. Moreover in the future, interventional research should be conducted to improve the current rate of adherence.

## Background

The World Health Organization (WHO) has produced and endorsed a partograph with the intention to improve labour management and reduce maternal and fetal morbidity and mortality [1]. The first obstetrician to describe the progress of labour graphically was Friedman (Friedman 1954) using a graphico-statistical analyses which measured the average rate of cervical dilation in labour [2]. Building on Friedman’s original work, Philpott R and Castle W were the first to describe a partograph which recorded all the labour ward observations on a single sheet and developed alert and action line which provided a clinical management tool [3, 4].

Partograph is an inexpensive graphically presenting tool in which labour observations, involving fetal condition, maternal condition and progress of labour are recorded [1, 5]. The aim of this tool is to alert midwives to abnormalities in maternal or fetal well-being and progress of labour [5]. Moreover, it is a useful tool to prevent prolonged and obstructed labour [6, 7]. Even though Cochrane review was not certain on the effect of routine use of partograph to monitor labour [5], the multi-center trial in South East Asia showed a significant reduction in intra-partum still birth, prolonged labour, augmentation, emergency caesarean section and vaginal examination [8]. WHO through its guideline also recommends applying this tool in all laboring mothers to identify deviations and institute interventions [1, 9]. Developing countries are not utilizing and recording partographs as intended during the intra-partum period, despite these regions having significant maternal and neonatal mortality related to prolonged and obstructed labour [1, 10–12]. Obstructed labour is the major cause of perinatal morbidity and mortality in Ethiopia. This can lead the woman to experience multiple complications like uterine rupture, postpartum hemorrhage, exhaustion and infection [1, 6, 7, 13].

A document review study conducted in Malawi showed that maternal blood pressure (BP) in 627(58.6%), temperature in 699(65.3%), molding in 272(25.4%), fetal heart rate (FHR) in 159(14.9%), cervical dilation in 24.5% and decent in 128(12%) of partographs were not recorded to the standard [14]. Other studies from Africa, including Ethiopia also reported that the quality of the partograph was poor and many of the parameters were partially recorded [10, 15, 16, 10, 17]. The overall completion of the parameters was 8.9% in Tanzania, 25.6% in Ghana and 8.8% in Uganda [11, 18, 19].

While a partograph on its own doesn’t address all aspects of quality of care, it can play a significant role in the time detection of labour complications [1]. The Government of Ethiopia in collaboration with its stakeholders has heavily intervened in maternal health through training of health care providers with Basic Emergency Obstetric and Newborn Care (BEmONC) which includes partograph use training [20]. However, maternal mortality which is estimated at 412 per 100,000 live births is still unacceptable in the country. This needs additional intervention [21].

There is no adequate evidence in relation to factors associated with the level of adherence in partograph use. However, knowledge, availability of partograph, shortage of staff, level of facility, qualification, professional difference, managerial support, staff motivation, training, and experience of Health Care Providers (HCPs) were some of the few identified associated factors in relation to partograph utilization [22–27]. Furthermore, there is no tangible study conducted at national level to address this research problem that encompasses all levels of health facilities, both private and public facilities with the desirable sample size. Moreover, proper record–keeping and consistent use of partograph will help efforts aimed at improving the quality of childbirth care and the 2016 National Emergency Obstetrics and Newborn Care (EmONC) survey [28] will provide a unique opportunity to address the information gap regarding partograph adherence. Therefore, this study aimed to assess the level of adherence and associated factors of partograph use in Ethiopia.

## Methods

### Data source

**Fig. 1:**
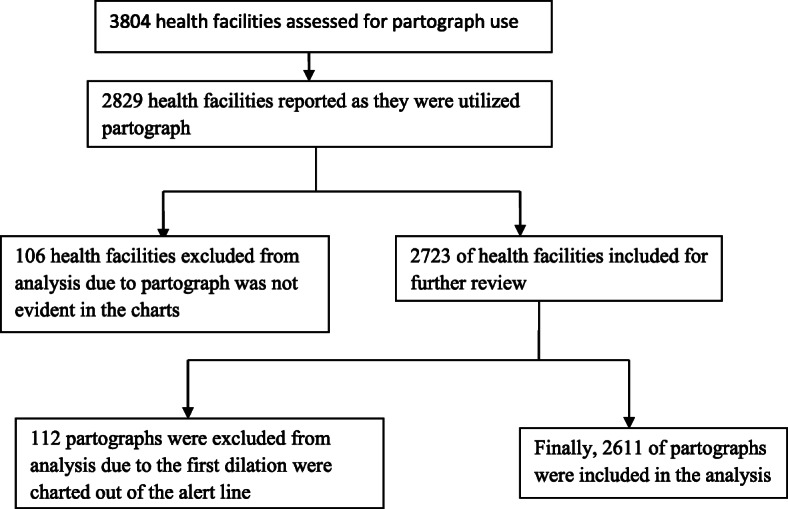
Flow diagram that shows partograph selection criteria for analysis, EmONC, 2016 Ethiopia

We used data from the EmONC assessment that was conducted in 2016 [28]. The EmONC assessment was a national cross-sectional census of health facilities, both at public and private health facilities that provided maternal and newborn health services. A total of 3804 facilities (293 hospitals, 3,459 health centers and 52 clinics) encompassing both government and private health facilities that offered delivery services throughout all regions of the country were assessed. Data from registers and birth records for the last 12 months prior to the survey were also extracted. In each facility, two latest charts of partograph were reviewed however two of them were not always completed. Therefore, for the purpose of this analysis we chose the partograph category with high frequency for the first dilation in active phase charted on the alert line correctly to represent the facilities. Thus, after a thorough exclusion criteria a total of 2,611 charts were assessed with regard to the completion of all components of the partograph and associated factors (Fig. 1). As eligibility criteria, partographs with first dilatation charted correctly on alert line were included in the final analysis.

### Measurements

Our primary outcome of interest was full adherence to standard partograph recording, using nine parameters/components of the partograph. It had a binary outcome: Adhered or not adhered to the standard recording. Therefore, a partograph was considered adherent to the standard if the nine parameters of the partograph were recorded completely as per the WHO standard. If at least one component was not recorded as per the WHO partograph, the partograph was considered non adherent. Provider-level characteristics: socio-demographic variables, qualification, level of training and work experience and facility characteristics: facility type, location and managing authority were the explanatory variables were included in this study.

### Statistical analysis

The analysis was performed using SPSS version 22™ software. Descriptive analysis such as frequency, percent, mean/median, and standard deviation were computed and the results were presented using text, tables and figures. Logistic regression model was used to assess the association between the predictor and outcome variable. Variables with p-value of < 0.25 were considered for inclusion in the multivariable logistic regression model. Finally, variables with p-value of < 0.05 in the multivariable analysis were considered to declare statistical significance. Odds ratio along with 95% confidence interval was computed to ascertain the strength of association between independent and dependent variables.

### Ethical issues

The primary researchers of the 2016 Ethiopia EmONC survey obtained ethical clearance from Ethiopian Public Health Institute (EPHI) and letter of permission from Federal Ministry of Health (FMOH). Therefore, there was no need for ethical clearance for this secondary analysis. However, permission to access the data was obtained from FMOH of Ethiopia.

## Results

### Socio-demographic characteristics of health care providers

The median age of participants was 25 years old (IQR = 3) ranging from 19 to 56 years old. About 1720 (65.9%) of service providers were female by sex. The service year of participants ranged from below 1 year up to 30 years. About 690 (26.4%) of the care providers had work experience below 1 year [Table 1].

**Table 1:** Socio-demographic characteristics of health care providers in Ethiopia, 2016, (*N* = 2611)

**Characteristics**	**Frequency**	**Percentage**
**Age of participants (years)**		
20–24	1102	42.2
25–29	1258	48.2
30+	250	9.6
**Participants level of qualification**		
Degree	423	16.2
Diploma	2187	83.8
**Types of profession **(*n = 2610)*		
Midwife	2320	88.9
Nurse	227	8.7
Health officer	63	2.4
**Year of experience (***n*** = 2610)**		
≤ 3 years	2237	85.7
4–6 years	306	11.7
7–9 years	34	1.3
≥ 10 years	33	1.3
**Received on-job training in maternal or newborn health**		
Has never received	459	17.6
In the last 6 months	1173	44.9
In the last year	480	18.4
More than a year ago	495	19
Refused	3	0.1

### Health facility characteristics

Availability of partograph forms were reported in 2,472 (94.7%) of the health facilities. Respondents were asked if there was any other document they fill in for a woman in labour and after birth in addition to partograph. Hence, 1293 (49.5%) of them reported they were utilizing ANC card, 1086 (41.6%) clinical case file, 165 (6.3%) administrative financial file and 6 (0.2%) other documents like admission sheet, lab request and delivery registration. Respondents were also asked which document they use when they have to prioritize. Hence, 1148 (68.3%) of them prioritized ANC card, 522 (31%) clinical case file, 6 (0.4%) administrative financial file and the rest 6 (0.4%) were other documents [Table 2].

**Table 2:** Characteristics of the health facilities in Ethiopia, 2016, (*N* = 2611)

**Characteristics**	**Frequency**	**percentage**
**Types of partograph used reported**		
Modified type of WHO partograph	2254	86.3
Simplified type of WHO partograph	31	1.2
Composite type of WHO partograph	49	1.9
^ a^Other types	4	0.2
Didn’t mentioned any of them	273	10.4
**Managing authority**		
Public facilities	2545	97.5
NGO-not-for –profit	14	0.5
Private-for-profit	23	0.9
Mission/faith based facilities	29	1.1
**Facility location**		
Urban	1119	42.9
Rural	1492	57.1
**Staffs trained for partograph(***n = 2610)*		
Yes	2282	87.4
No	328	12.6
**Partograph used in the last 3 months**		
Yes	2519	96.6
No	90	3.4
**Reasons why not used in the last 3 months (***n*** = 90)**		
Shortage of staff	4	4.4
Lack of training	6	6.7
Interruption of partograph forms	64	71.1
Weak management	10	10.9
Lack of supportive policy	4	4.3
There were no clients	10	10.9
**staff rotation in maternity care unit (***n = 2610)*		
Yes	876	33.6
No	1734	66.4
**Availability of labour management protocol**		
Reported as not available	1092	41.8
Reported and observed	1311	50.2
Reported but couldn’t find	208	8

^a^Other types: follow up labour progress recording sheet and lab request papers

### Quality of the partograph reviewed

Even though 3801 health facilities were involved in the BEmONC survey, only 2611 of health facilities were eligible for partograph review. Among the 2611 partographs reviewed, 561(21.5%, CI: 19.91, 23.06) of them were recorded to the standard based on the WHO guidelines. The median time of hours and minutes elapsed between first exam and delivery was 4:0 (IQR 4:20) hours. The median of FHB at admission was 140(IQR = 10) beat per minute. Majority, 2166 (83%) of the partographs were filled in as labour progressed, while 445 (17%) were judged to have been filled after delivery. Apgar score was not filled in about 586 (22.4%) of cases [Table 3].

**Table 3:** Level of the recording of the parameters of partograph in Ethiopia, 2016, (*N* = 2611)

**Parameters**	**Frequency**	**Percentage**
**Progress of labour**		
Plotted descent of head every 4 hours		
Yes	1651	63.2
Plotted uterine contraction every 30 minutes		
yes	2468	94.5
Plotted cervical dilation every 4 hours		
Yes	2340	89.6
**Maternal condition**		
Plotted maternal temperature every 2 hours		
Yes	1385	53
Plotted maternal blood pressure every 4 hours		
Yes	2102	80.5
Plotted maternal pulse rate every 30 minutes		
Yes	1841	70.5
**Fetal condition**		
Plotted fetal heart rate every 30 minutes		
Yes	2437	93.3
Plotted molding of the fetal skull, at every 4 hours		
Yes	1308	50.1
Plotted color of liquor every per vaginal examination(at least 4 hours apart)		
Yes	1842	70.5

Outcome of the baby and other information recorded on the partograph

Majority, 2379 (91.1%) of the delivery were normal live births, 17(0.7%) live births with birth asphyxia and 18(0.7%) were still births. However, in 197 (7.5%) of the partographs, information with regard to the birth outcomes was not recorded. The Median of hours passed to the right of action line in those who delivered on or to the right of action line was 3:30(IQR = 7:43, *n* = 49). Referral and other activities performed during the course of partograph recording are detailed in Table 4.

**Table 4:** Recording of information on the partograph related to referral and augmentation in Ethiopia, 2016, (*N *= 2611)

**Characteristics**	**Frequency**	**Percentage**
**The woman referred from another facility**		
No/no information	2556	97.9
Yes	55	2.1
**When the woman referred (***n*** = 55)**		
On or left of the alert line	21	38.2
Between the alert and action lines	1	1.8
On or to the right of the action line	1	1.8
Could not be determined	32	58.2
**According to the partograph when did the woman deliver**		
On or left of the alert line	1990	76.2
Between the alert and action lines	391	15
On or to the right of the action line	61	2.3
Information not recorded	169	6.5
**Augmentation used**		
Yes	112	4.3
No	2499	95.7
**When was augmentation used(***n*** = 112)**		
Not recorded	61	54.5
On the alert line	32	28.5
Between the alert and action line	14	12.5
On or beyond the action line	5	4.5
**Time at delivery filled in**		
Yes	2216	84.9
No	395	15.1
**Mode of delivery**		
Spontaneous vertex delivery	2471	94.6
Vacuum/forceps delivery	14	0.5
Cesarean delivery	9	0.3
No information	117	4.5

**Fig. 2:**
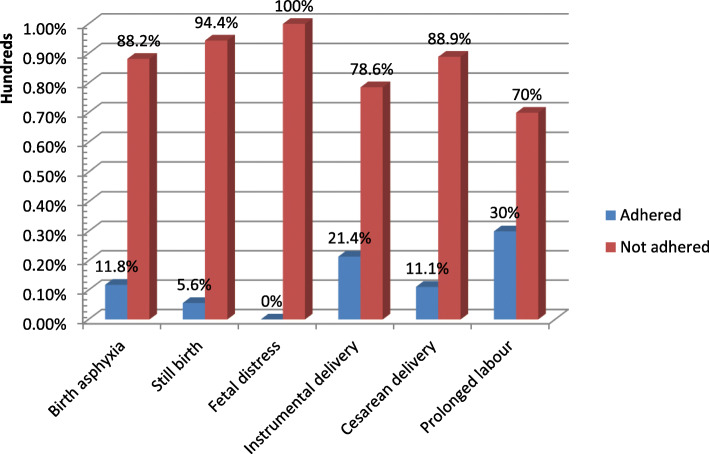
The relationship of health care professionals’ adherence to partograph use and labour outcomes, EmONC 2016, Ethiopia

A cross-tabulation analysis was done between level of partograph adherence and its outcomes. Therefore, the distribution of the birth outcomes and mode of delivery between the partographs completely and partially recorded is presented in Fig. 2.

### Factors associated with adherence to partograph use

At -bivariate analysis, facility location, level of EmONC, experience of the provider, type of facility, managing authority and providers’ level of education were found to be significantly associated with adherence to partograph use. After multivariate analysis, only location of the health facility was found to be significantly associated with the standard of partograph recording. Hence, the odds of adherence to the standards of partograph record was 1.4 in health facilities located in rural areas when compared to health facilities located in urban areas (AOR = 1.44; 95% CI:1.15, 1.80, ***P-Value*** 0.002) (Table 5).

**Table 5:** Factors associated with level of health care professionals’ adherence to partograph use in Ethiopia, 2016, (*N* = 2611)

**Variables**	**Adherence of partograph**		**COR (CI = 95%)**	**AOR (CI = 95%)**	***P*-value**
	**Yes, n (%)**	**No, n (%)**			
**Residence**					
Urban	201(18)	918(82)	1	1	
Rural	360(24.1)	1132(75.9)	1.51(1.02, 2.22)	**1.44(1.15, 1.80)**	**0.002**
**Level of EmONC**					
BEmONC	424(22.1)	1492 (77.9)	1.51(1.02, 2.22)	1.19(0.53, 2.70)	
CEmONC	33(15.9)	175(84.1)	1	1	
**Work experience**					
<=5 year	544(21.8)	1948(78.2)	1.78(1.04, 3.04)	1.66(0.91, 3.02)	
>=6	16(13.6)	102(86.4)	1	1	
**Type of facility**					
Hospital	36(15.3)	199(84.7)	1	1	
HC/MCH	525(22.1)	1851(77.9)	1.57(1.09, 2.27)	0.94(0.42, 2.11)	
**Managing authority of the health facility**					
Government/Public	554(21.8)	1991(78.2)	2.35(1.07, 5.16)	1.50(0.62, 3.62)	
NGO	7(10.6)	59(89.4)	1	1	
**Level of degree**					
Degree	72(17)	351(83)	1	1	
Diploma	488(22.3)	1699(77.7)	1.40(1.07, 1.84)	1.14(0.84, 1.55)	

*COR *Crud odds ratio; *AOR *Adjusted odds ratio

## Discussion

This study used data from the 2016 EmONC assessment in Ethiopia which is the first survey to capture detailed information on partograph adherence of health care providers at national level. In this paper we discuss the overall level of adherence of partograph, each component of the partograph, the perinatal outcomes and factors associated with adherence of partograph recording in Ethiopia. We also discuss the implication of the study findings for program improvement and future research in Ethiopia.

Our study showed that the overall adherence of health care professionals to all the components of the partograph as per WHO guidelines was poor (one out of five charts were completed as per the WHO standard). More adverse outcomes were reported when partographs were not completed based on the WHO guideline. Health care service providers working in rural health facilities were more likely to fill the components of the partograph according to the WHO guideline when compared to their counterparts.

Specifically, 50% of molding, 70.5% color of liquor status and 93.3% of fetal heart beat (FHB) were recorded to the standard on partograph. This is consistent with the studies conducted in Bangladesh and many African countries including Ethiopia where, the parameters of the partograph were recorded partially against the WHO guideline [15, 11, 14, 16, 29, 30, 29, 30, 10, 17, 23] even though there were slight differences in the degree of completion between these studies. This low level of completion implies that monitoring of fetal condition using partograph was insufficient while it is essential to pick up fetal complications early and thus institute interventions. In order to have good fetal outcomes, fetal condition should be monitored and recorded to the standard.

In the current study, about 89.6%, 63.2%, and 94.5% of the cervical dilation, descent and uterine contraction respectively were recorded to the standard. The current recorded cervical dilation is nearly comparable with the findings reported from Bangladesh and Malawi, where 70% and 75.5% of the partographs respectively were recorded to the standard [15, 14]. However, low levels of record (55.8%, 32.9% and 31.8%) on cervical dilation were reported in studies conducted in Tanzania and Ethiopia respectively [11, 10, 17].The observed discrepancy could be due to sample size difference among these studies. The current study conducted at national level encompassing all levels of health facilities with large sample size, whereas those studies were local studies with a relatively small sample size. On the other hand, studies conducted in Ghana and Zambia revealed that the reviewed partographs fully documented the dilation of cervix [16, 30]. This could be due to that health care service providers in Ghana and Zambia might give extra attention to cervical dilation to measure the progress of labour irrespective of the other parameters. The observation that partial completion of partographs on descent of fetal head and uterine contraction in the current report were similar with studies conducted in Ghana and Zambia [16, 30]. However, low level of record on uterine contraction and descent of the fetal head were reported from Malawi (38.9% and 46% [14]), Uganda (62.8% and 62%) [29], Tanzania (14.6% and 59.9%) [11], Bangladesh (77.8% and 68.5%) [15] and Ethiopia (Addis Ababa: 20.7% and 16%; Bale Zone: 23.8% and 7.1%) [10, 17] respectively. This suggests that health care providers give less attention to the progress of labour, which might result in missed diagnosis of obstructed and prolonged labour.

Using partographs to monitor labour progress is effective in reducing obstructed and prolonged labour, which is a major direct cause of maternal and newborn morbidity and mortality. These complications can lead to ruptured uterus, postpartum hemorrhage, infection, obstetric fistula and fetal injury or death [6, 7].

The present study also found that vital signs of the mother were partially completed. About 80.5%, 70.5%, and 53% of the blood pressure (BP), pulse rate (PR) and temperature respectively were recorded, somewhat less than the Zambian study where 96.8% of BP and 85.6% of PR were completely recorded [30]. The record of BP and temperature were also consistent with study conducted in Ghana [18]. On the other hand, the current record of maternal vital signs is high when compared to reports from Uganda (37.2%, 28.2% and 11.8%) [29], Tanzania (23.6%, 43.8%, and 22.3%) [11] and Bangladesh (67.6%, 3.2%, and 32.4%) [15] respectively. It was also high as compared to studies conducted in Malawi and Ethiopia [14, 10, 17, 23].The discrepancy could be due to setting, time and sample size difference. It could be also due to shortage of vital sign equipments in some health facilities. Monitoring the mother’s vital signs during the intra-partum period is crucial for identifying complications and instituting timely interventions. For instance, checking of BP and temperature may help to identify pre-eclampsia/eclampsia and sepsis respectively, which are among the direct causes of maternal mortality globally [31, 32]. Particularly in Ethiopia, hypertensive disorders of pregnancy and sepsis are leading causes of maternal mortality, accounting for 16.9% and 14.7% of deaths respectively [32].

The overall average of adherence to partograph use was comparable only with one study from Ghana, where 25.6% of the partographs were completely documented based on the WHO guideline [18]. However, the current report is higher when compared to studies conducted in Tanzania and Uganda where, 8.9%and 8.8% of the partographs respectively were completely recorded to the standard [11, 29]. Even though there are no tangible studies conducted in the country to compare with the current findings, our sample size likely overestimates the rate of adherence as compared to the above two studies. The discrepancy could be due to difference in setting, sample size and level of professional competency among the countries. Moreover, it could also be due to health worker acceptability, health system support, effective referral system, human resources and health provider competence. These were identified as possible challenges for consistent and complete use of partograph in some studies [1, 33]. In the past two decades, the Government of Ethiopia in collaboration with stakeholders invested significantly in maternal and child health care services by increasing availability of midwives, training of midwives and mentoring and supervision. Yet despite these notable interventions, the current finding still remained below the standard [34]. Probably, the partial recording of the parameters partly explains the existing high maternal and perinatal mortality in Ethiopia [21].

We did not conduct regression analysis examining the relationship of partograph adherence to adverse perinatal outcomes, as the relatively low frequency of adverse perinatal outcomes did not allow for robust modeling. However, in the cross tabulation analysis most of the adverse outcomes were found to be recorded among the partially completed partographs. Evidence about the frequency of adverse perinatal outcomes and partograph completion are inconsistent across the literatures. For instance, studies conducted in Ghana, Nepal, and Tanzania revealed that birth asphyxia, intrapartum stillbirths and low Apgar score were found to be significantly associated with incompletely recorded partographs [16, 19, 35]. The WHO multicenter trial also confirmed that use of partograph during labour reduces prolonged labour, augmentation, emergency caesarean sections and intrapartum stillbirths [8]. However, the Cochrane review on partograph use found that perinatal outcomes were not significantly improved with use of a partograph compared to labour monitored without a partograph [5]. Poor quality of monitoring during labour which might result into missed diagnosis of maternal and fetal complications and institute interventions. However, confounding factors like chronic diseases of the mother and poor skill of the health care providers can also contribute in the occurrence of adverse perinatal outcomes.

Health care professionals working in rural health facilities were more likely to adhere to the standards of partograph completion when compared to their counterparts. This could be due to low number of deliveries attended in rural health facilities. Whereas labour wards found in under-resourced urban settings like Ethiopia might be busy as a result health professionals would be overloaded and couldn’t monitor all women appropriately using the partograph. Moreover, 57% of the facilities included in this study were rural facilities. On the other hand, urban health facilities might be equipped with more experienced health workers, capable in managing emergencies and smooth referral abilities compared to rural facilities. As a result, health workers might have over confidence and overlook the strict use of partograph to monitor the progress of labour. Mentoring and supervision from regional health bureaus and stakeholders also might have focused on rural health facilities.

The significant strength of this study was the use of a large sample size to ensure for the reliability of the sample mean. However, this study had some limitations. First, the data were collected through retrospective chart review. This might not necessarily reflect the actual monitoring of labour progress using partograph. This is evidenced by some of the partographs in this study and elsewhere that were judged to be recorded retrospectively. Second, the assessment was based on data extracted from charts and registers that are often incomplete. For example, urine test was absent which is part of the parameters of the partograph. Third, this study assessed only documentation of the parameters to monitor the progress of labour but we could not make sure that whether the partograph documentation was translated in to labour management and decision making.

## Conclusions

This study revealed that adherence of partograph use in Ethiopia is poor. Molding, maternal temperature and decent were the least recorded among the parameters of the partograph. This implies there is a poor quality of intra-partum care in health facilities which could be partly the reason for the high maternal and perinatal morbidity and mortality in Ethiopia. Facilities located in rural areas adhered more to implement partograph use. Thus, to ensure quality of intra-partum care, we recommend urban health facilities to have strong supervision and mentoring periodically. In addition, daily auditing strategies and onsite training of midwives should be introduced mainly for the urban health facilities. Moreover, interventional research should be conducted to improve the rate of adherence on partograph use in Ethiopia.

## Data Availability

The database used and/or analyzed during the current study are available from the corresponding author on reasonable request because of the database is large which includes other maternal health related data.

## Abbreviations

ANC: Antenatal care
BP: Blood pressure
EDHS: Ethiopian demographic health survey
EmONC: Emergency obstetric and newborn care
EPHI: Ethiopian public health institute
FHB: Fetal heart beat
FMoH: Federal ministry of health
IQR: Inter quartile range
MCH: Maternal and child health
NGO: Non-governmental organization
PR: Pulse rate
WHO: World health organization
